# Incidental Detection and Management of a Neuroendocrine Carcinoid Tumor of the Appendix in a Young Male Patient

**DOI:** 10.7759/cureus.44386

**Published:** 2023-08-30

**Authors:** Anjani H Turaga

**Affiliations:** 1 Medicine and Surgery, Gandhi Medical College, Hyderabad, IND

**Keywords:** tumor appendix, laproscopic appendectomy, neuroendocrine carcinoma of appendix, carcinoma of the appendix, appendix

## Abstract

The case presented is of a male patient in his 20s who initially underwent an appendectomy for suspected acute appendicitis. However, histopathological examination revealed the unexpected diagnosis of a neuroendocrine carcinoid tumor of the appendix. A subsequent right hemicolectomy was performed for complete tumor removal.

## Introduction

Neuroendocrine tumors (NETs) of the appendix are rare neoplasms, accounting for approximately 0.3-0.9% of all appendix tumors [1,2]. These tumors are often incidentally discovered during appendectomy or investigations for abdominal pain or appendicitis [2]. While most neuroendocrine appendiceal tumors have an indolent clinical course and a favorable prognosis, some can metastasize to regional lymph nodes or distant sites [1].

The appendix, traditionally considered a vestigial organ without significant physiological function, has recently been recognized for its potential role in the immune system, acting as a reservoir for beneficial gut bacteria [3]. Despite this newfound appreciation, the appendix can still be affected by various pathologies, including inflammations, infections, and neoplasms.

NETs arise from neuroendocrine cells located throughout multiple organ systems, including the gastrointestinal tract, respiratory system, pancreas, and adrenal glands [4]. These tumors are characterized by their capacity to secrete hormones and bioactive substances, resulting in a wide range of clinical manifestations and diagnostic challenges.

Appendiceal NETs can be classified into three subtypes, namely, classic carcinoid tumors, goblet cell carcinoids (GCCs), and mixed adenocarcinoma-neuroendocrine tumors (MANETs) [1]. Classic carcinoids are well-differentiated tumors without mucin production and have a low malignant potential. GCCs, on the other hand, exhibit both neuroendocrine and mucin-producing goblet cells, leading to a more aggressive clinical course. MANETs represent hybrid tumors composed of neuroendocrine and glandular components, with varying malignant potential determined by the proportions of each component [5].

Diagnosing appendiceal NETs can be challenging, as they often lack specific symptoms or may present with nonspecific complaints such as abdominal pain, bloating, or changes in bowel habits [2]. Due to the rarity of these tumors, clinicians may have a low index of suspicion, resulting in delayed diagnosis and treatment. Consequently, these tumors are frequently incidentally detected during appendectomy or imaging studies performed for unrelated reasons.

Imaging modalities such as ultrasound, computed tomography (CT), and magnetic resonance imaging (MRI) can aid in the detection and characterization of appendiceal tumors. Notably, MRI with diffusion-weighted imaging (DWI) has shown promising results in identifying and distinguishing appendiceal tumors, including NETs [3]. This noninvasive technique provides valuable information regarding tumor size, location, and potential invasion of adjacent structures, facilitating surgical planning and prognostic evaluation.

Management of NETs of the appendix generally involves surgical removal of the appendix, with the extent of surgery guided by tumor characteristics and the presence of metastasis [5]. Localized, nonmetastatic tumors can be adequately treated with appendectomy alone, which is often curative. However, larger tumors invading adjacent structures or associated with lymph node metastasis may require right hemicolectomy or segmental resection to ensure complete tumor resection and regional lymph node clearance [5].

In some cases, adjuvant treatments such as chemotherapy or somatostatin analogs may be recommended, particularly for advanced disease or tumors with high-risk features such as lymphovascular invasion or positive lymph nodes [5]. Decisions regarding adjuvant therapy are made through a multidisciplinary approach, considering tumor grade, stage, and individual patient factors.

In this case report, we present the incidental detection and management of a neuroendocrine carcinoid tumor of the appendix in a young male patient. Our report aims to underscore the significance of early diagnosis and appropriate management of such tumors, particularly in young patients who may have a longer life expectancy and an increased risk of metastatic spread. By sharing this case, we hope to raise awareness among clinicians and encourage timely recognition and intervention for similar cases in the future.

## Case presentation

A young male patient, in his late 20s, presented to the emergency department with a history of worsening abdominal pain localized to the right lower quadrant for the past 24 hours. He also experienced episodes of nausea and vomiting but did not report any other symptoms such as changes in bowel habits, unexplained weight loss, or similar episodes in the past. On physical examination, tenderness at McBurney’s point with guarding was observed, consistent with acute appendicitis. Laboratory investigations revealed an elevated white blood cell count and C-reactive protein level, while other parameters were within normal limits. An abdominal ultrasound showed an enlarged appendix that was non-compressible, along with the presence of free fluid, supporting the diagnosis of acute appendicitis.

The patient was informed about the diagnosis and the need for surgical intervention, specifically a laparoscopic appendectomy. Informed consent was obtained, and the procedure was successfully performed without any intraoperative complications. The excised appendix, upon histopathological examination, revealed an unexpected finding of a neuroendocrine carcinoid tumor situated in the mid-appendix, measuring 1.5 cm in its largest dimension. The resection margins were clear, indicating complete removal of the tumor. However, due to the presence of the neuroendocrine carcinoid tumor, further imaging investigations were conducted to evaluate for possible metastasis. A CT scan of the abdomen and pelvis, along with an octreotide scan, showed no evidence of distant metastasis or lymphadenopathy.

**Figure 1:**
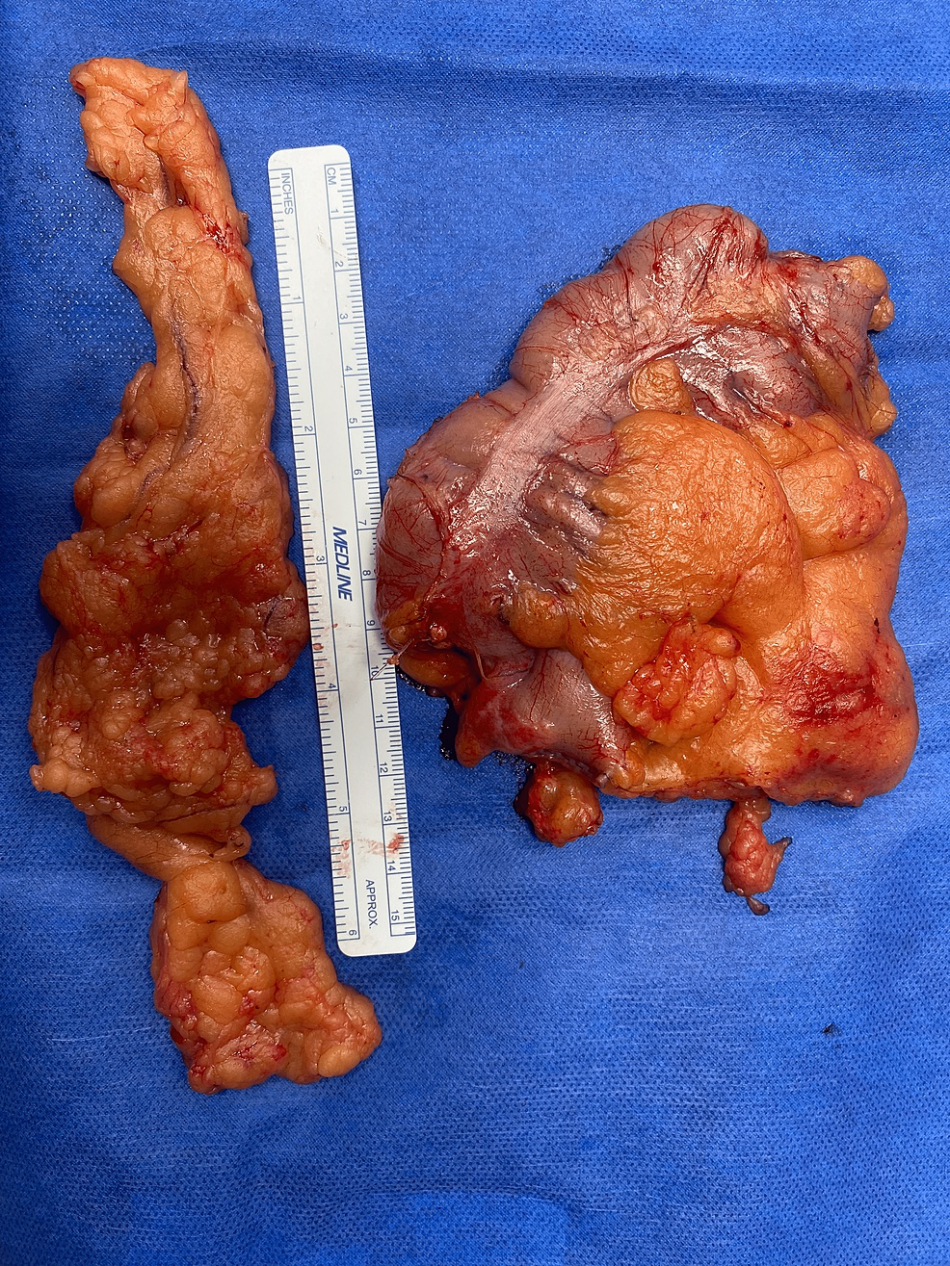
On the left, appendix with clean margins. On the right, the hemicolectomy specimen with the tumor.

Considering the size of the tumor and after extensive discussions with the patient regarding the potential risks and benefits, it was decided to proceed with a right hemicolectomy to ensure complete excision of any residual tumor tissue. The right hemicolectomy procedure was performed without any complications, and the patient’s recovery in the postoperative period was uneventful. A picture of the specimen is shown in Figure 1.

**Figure 2:**
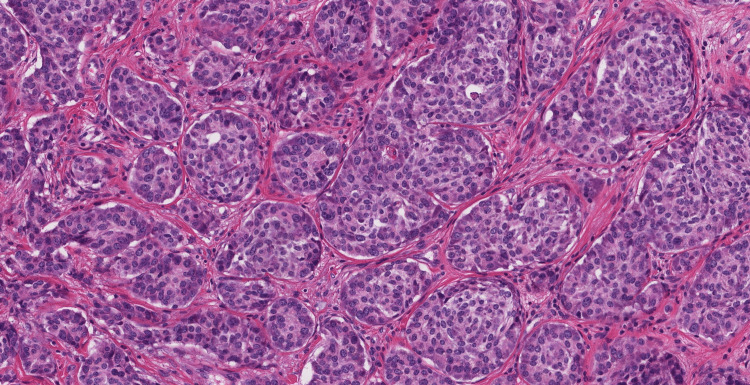
Histopathological image showing well-differentiated neuroendocrine tumor of the appendix.

Following the right hemicolectomy, the resected specimen was sent for further histopathological examination. The examination confirmed the presence of a neuroendocrine carcinoid tumor within the appendix, with clear resection margins, indicating successful removal of the tumor. The tumor was found to be located in the mid-appendix and measured 1.5 cm in its largest dimension. Microscopic analysis revealed the characteristic features of a well-differentiated neuroendocrine carcinoid tumor, including nests and trabeculae of uniform tumor cells with round-to-oval nuclei and finely granular chromatin. Mitotic activity within the tumor was low, and there was no evidence of lymphovascular invasion. Figure 2 shows the histopathological image of the tumor.

Given the unexpected finding of a neuroendocrine carcinoid tumor in a young patient, further discussions were held with the patient to understand his medical history, family history, and any potential risk factors for the development of this tumor. The patient denied any significant past medical history, including any previous gastrointestinal symptoms. Family history was negative for any known genetic syndromes associated with NETs. Genetic counseling was recommended to the patient to assess the potential underlying genetic predisposition; however, the patient declined genetic testing at this time.

To assess the overall extent of the disease and evaluate for any residual tumor or possible metastasis, close postoperative follow-up was planned. This included regular physical examinations, measurement of tumor markers such as chromogranin A and serotonin levels, and periodic imaging studies such as CT scans. The patient was also advised to report any new symptoms or changes in his health status promptly.

At the time of the report, the patient had completed his surgical treatment successfully and was recovering well. The multidisciplinary team, consisting of surgeons, oncologists, and pathologists, continued to monitor his progress and formulate a personalized follow-up plan based on his individual characteristics and tumor pathology. The aim was to ensure early detection of any potential recurrence or metastasis and to provide appropriate interventions to optimize the patient’s long-term outcomes.

## Discussion

Neuroendocrine carcinoid tumors of the appendix represent a relatively rare pathology, often discovered incidentally during appendectomies performed for suspected appendicitis, as was the case in our patient. These tumors are generally small and found in the distal third of the appendix, contrasting with our case where the tumor was located in the mid-appendix and measured 1.5 cm [5].

The management strategy for appendiceal neuroendocrine tumors is primarily dictated by the size of the tumor. Tumors measuring less than 1 cm can usually be managed with a simple appendectomy, given their low potential for metastasis. However, for tumors measuring between 1 and 2 cm, as in our case, the decision for additional surgery is guided by factors such as age, general health of the patient, and tumor characteristics [4].

Our patient underwent a right hemicolectomy given the tumor size and location. This decision aligns with the European Neuroendocrine Tumor Society guidelines that suggest right hemicolectomy for tumors greater than 2 cm or for those with involvement of the base of the appendix, lymphovascular invasion, or high-grade tumors [5].

Comparison to the existing literature reveals that our case mirrors the typical presentation and management strategies for appendiceal carcinoid tumors, with the exception of tumor location. This case underscores the importance of histopathological examination of the appendix, even in clinically straightforward cases of acute appendicitis. It also emphasizes the need for a multidisciplinary approach in managing such incidental findings to ensure optimal patient outcomes.

Despite the relative rarity of appendiceal NETs, the incidence appears to be rising, which might be due to the increased use of imaging and endoscopy as well as more frequent histopathological examination of appendectomy specimens [1].

Once diagnosed, these tumors carry a generally favorable prognosis, particularly when identified at an early stage and managed appropriately. The five-year survival rate for localized appendiceal carcinoid tumors is reported to be more than 90% [2]. In our case, the patient’s postoperative period was uneventful, and the subsequent follow-up visits did not show any evidence of disease recurrence or metastasis. He remains under regular surveillance according to the guidelines.

This case report highlights the importance of a high index of suspicion for this rare condition when dealing with patients presenting with signs and symptoms of acute appendicitis. It also underscores the value of routine histopathological examination of the appendix following appendectomy to rule out such incidental findings.

Learning points

Neuroendocrine carcinoid tumors of the appendix, though rare, can present as acute appendicitis. Unexpected histopathological findings post-appendectomy require further evaluation and potentially additional surgical intervention. Despite their potential for metastasis, the prognosis of localized carcinoid tumors is generally favorable with appropriate surgical management.

Patient’s perspective

The diagnosis of a neuroendocrine carcinoid tumor was a complete surprise to me. I had initially come to the hospital with what I thought was a typical case of appendicitis. However, the news that I had a tumor was quite shocking, as I am generally a healthy person with no significant medical history.

Once the initial shock wore off, my doctors were very thorough in explaining the diagnosis, the nature of the tumor, and the potential risks and benefits of further surgery. They provided me with all the necessary information in a clear and understandable way, allowing me to make an informed decision about my treatment.

I chose to proceed with the right hemicolectomy. The postoperative period was challenging, but the constant support and reassurance from the healthcare team helped me through it.

In the following months, I felt relieved to learn that the tumor had been completely removed and there were no signs of disease recurrence. This experience has made me more aware of my health. I am now regularly attending follow-up appointments and maintaining a healthy lifestyle.

Looking back, I feel grateful for the quality of care I received. I hope my story can help others who might find themselves in a similar situation. It is important to remember that even in the face of unexpected diagnoses, there are teams of medical professionals ready to provide the best possible care.

## Conclusions

We presented a case of incidental detection and management of a neuroendocrine carcinoid tumor of the appendix in a young male patient. The unexpected finding of the tumor prompted further investigations, ultimately leading to the decision for a right hemicolectomy to ensure complete removal. Although neuroendocrine carcinoid tumors of the appendix are rare, this case highlights the importance of thorough examination and consideration of differential diagnoses in patients with atypical presentations of appendicitis. With appropriate management and regular follow-up, the patient has a favorable prognosis. Awareness of such cases is crucial to promote early diagnosis, optimize treatment strategies, and improve outcomes for patients with NETs of the appendix.
